# Sclerosing pneumocytoma in a 1-year-old girl presenting with massive hemoptysis: A case report

**DOI:** 10.1016/j.amsu.2021.01.004

**Published:** 2021-01-08

**Authors:** Albaraa Bara, Ibrahim Adham, Obada Daaboul, Fatima Aldimirawi, Bassam Darwish, Lina Haffar

**Affiliations:** a Faculty of Medicine, Damascus University, Damascus, Syrian Arab Republic; b Pediatric Pulmonologist Consultant, Damascus, Syrian Arab Republic; c Department of Thoracic Surgery, Al-Mouassat University Hospital, Damascus, Syrian Arab Republic; d Department of Pathology, Faculty of Medicine, Damascus university, Damascus, Syrian Arab Republic

**Keywords:** Case report, Sclerosing pneumocytoma, Sclerosing hemangioma, Hemoptysis, Child, Thoracotomy

## Abstract

**Introduction and importance:**

Sclerosing pneumocytoma (SP) is a rare benign neoplasm of the lung with peak age incidence in middle aged-women. Here we report, for the first time in the literature, a case of a 1-year-old girl with SP.

**Case presentation:**

A 1-year-old girl was reported to emergency department for massive hemoptysis. After admission, the patient had a three-days episode of melena, with normal body temperature and generally stable condition.

**Clinical discussion:**

Fiberoptic bronchoscopy was normal. MSCT was done along with angiography and Three-Dimensional Reconstruction which revealed a well-circumscribed round mass with well-defined borders located near the vessels in the upper lobe of left lung. Anatomic lingula resection was performed. Hilar node was also resected. The histopathological examination confirmed the presence of SP. Fourteen months postoperatively, the patient was in a good health with no clinical or radiological evidence of recurrence.

**Conclusion:**

SP is a rare benign tumor which usually presents in middle aged-women asymptomatically or with nonspecific symptoms. We report this case to highlight that SP should be considered in cases of hemoptysis in young children.

## Highlights

• Sclerosing Pneumocytoma could be asymptomatic or rarely present with hemoptysis.
• SP could be diagnosed in all ages including young children.
• Confirmed diagnosis of SP is made by histopathological study.
• Chest radiograph is non-specific in most SP cases.

## 1 Introduction

Sclerosing pneumocytoma (SP) is a rare pulmonary benign neoplasm of undefined origin. It was named at first sclerosing hemangioma [1]. Although its name implicated a vascular neoplasm, further studies have reported that it is a tumor of pneumocytic origin with a dual population of surface cells resembling type II pneumocytes and round cells. These cells have considerable multiple differentiation potential, and slightly different histogenetic profiles. For this reason, it has been classified under the more convenient name pneumocytoma according to World Health Organization (WHO) classification of lung and pleural tumors [2].

SP appears morphologically as a small, round, or oval homogeneous nodule with smooth margins. On dynamic computed tomography scan (CT scan), this lesion shows strong and rapid enhancement caused primarily by hemangiomatous or papillary components [3]. Although it is generally considered to be a benign tumor, it represents a diagnostic challenge due to its controversial etiology and biologic behavior, as well as the diversity of pathohistological findings. In addition to its rare overall incidence, it's extremely rare to occur in the child population [4]. It predominantly occurs in middle-aged women [5]. Here, we report a case of sclerosing pneumocytoma in one-year old girl presented with massive hemoptysis. This work has been reported in line with the SCARE criteria [6].

## 2 Case presentation

**Figure 1:**
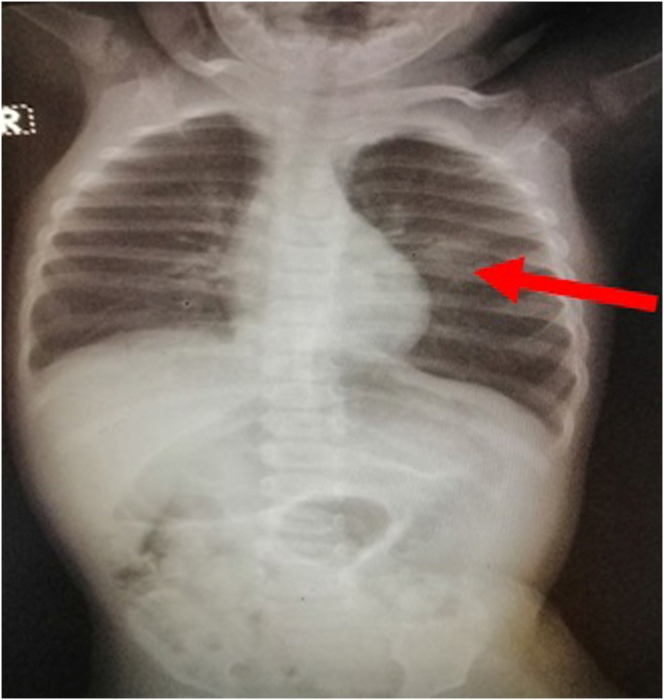
Patient's chest radiography. A round-shaped opacity in the left lung could be noted (arrow).

A one-year-old girl with no significant medical, surgery, or family history was brought to the emergency. Her parents reported blood coming out from her mouth. There were no significantly abnormal findings on physical examination. Laboratory tests including complete blood count (CBC), bleeding time (BT), prothrombin time (PT), activated partial thromboplastin time (aPTT), urine analysis, and arterial blood gas analysis were within normal ranges. Chest radiography revealed round-shaped opacity in the left lung (Fig. 1).

**Figure 2:**
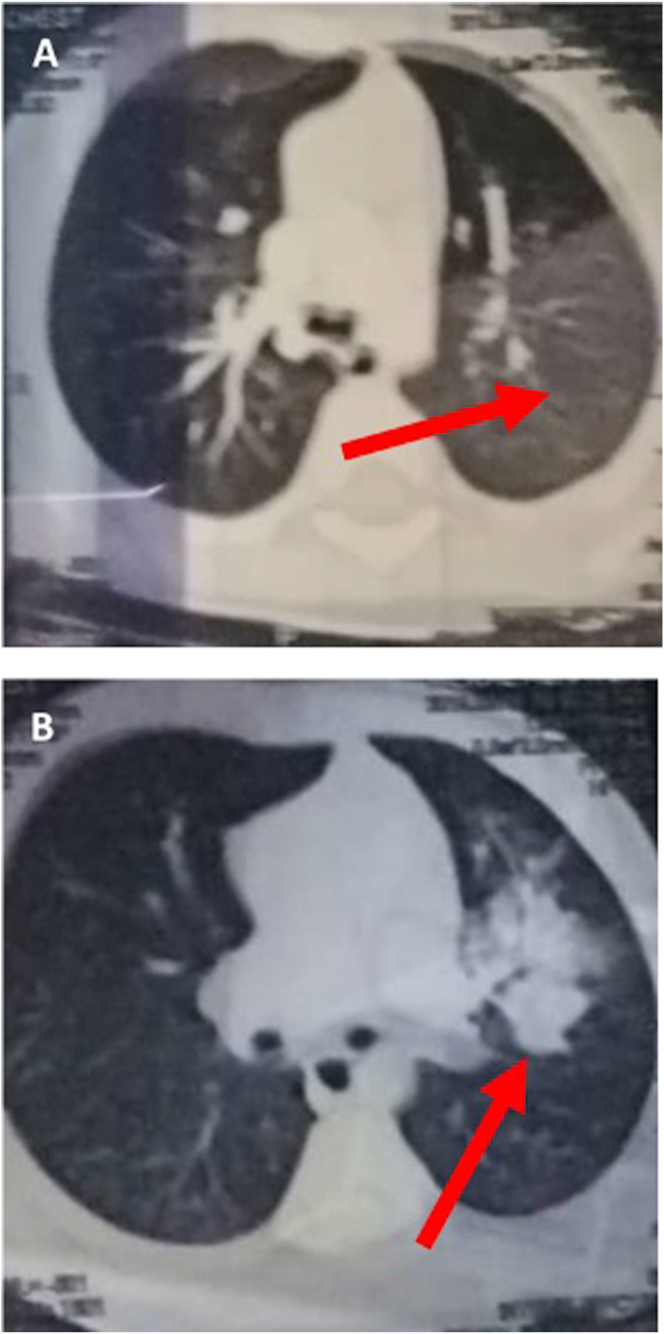
A. Contrast enhanced MSCT images showing left sided hemorrhagic ground-glass opacities (arrow). B. A well-circumscribed round mass with well-defined borders located in the upper lobe of left lung could be seen (arrow).

Four hours later, she developed massive hemoptysis with hemodynamic deterioration, as her hemoglobin levels suddenly decreased to 6.5 g/dl. After red blood cells transfusion, chest contrast enhanced multislice computed tomography (MSCT) was performed. It revealed areas of hemorrhagic ground-glass opacities in the left upper lobe and lingula (Fig. 2A), as well as a well-circumscribed round mass with well-defined borders and estimated diameter of 1.6 cm. The mass was located in the upper lobe of left lung (Fig. 2B).

**Figure 3:**
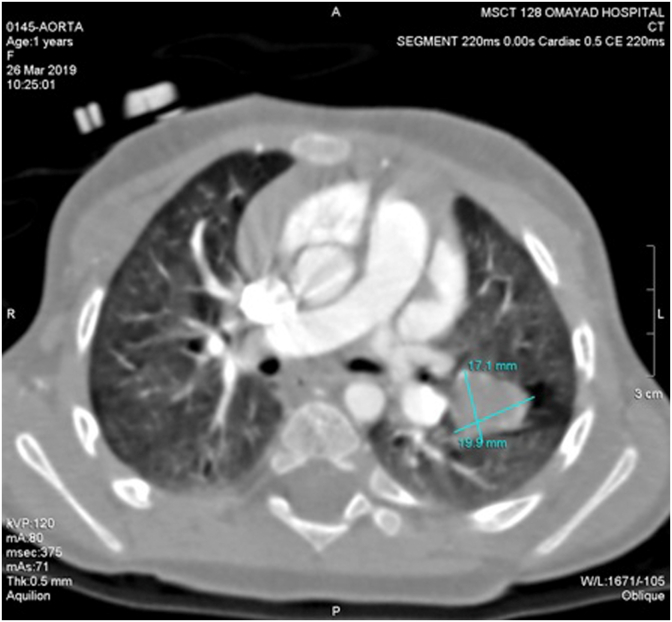
Contrast enhanced MSCT image showing a well-circumscribed round mass with well-defined borders and estimated dimensions of 2.0 × 1.7 cm located near the vessels in the upper lobe of left lung.

Thereafter, the patient was admitted for further monitoring. She had a three-days episode of melena, with normal body temperature and generally stable condition. She also underwent further assessment through fiberoptic bronchoscopy, which was normal.The MSCT was repeated along with angiography and Three-Dimensional Reconstruction which demonstrated resolving of pulmonary hemorrhage and normal pulmonary arteries and aorta, with no signs of pulmonary sequestration or abnormal vessels. It also revealed a well-circumscribed round mass with well-defined borders and estimated dimensions of 2.0 × 1.7 cm located near the vessels in the upper lobe of left lung, without clear vascular or bronchial connection (Fig. 3).

She underwent a left posterolateral thoracotomy. Anatomic lingula resection was performed. Hilar node was also resected. The surgical procedure went without any complication.

The resected mass measured 2 cm in its largest diameter. It was well circumscribed, with white-pink color. The hilar node measured 0.7 cm.

**Figure 4:**
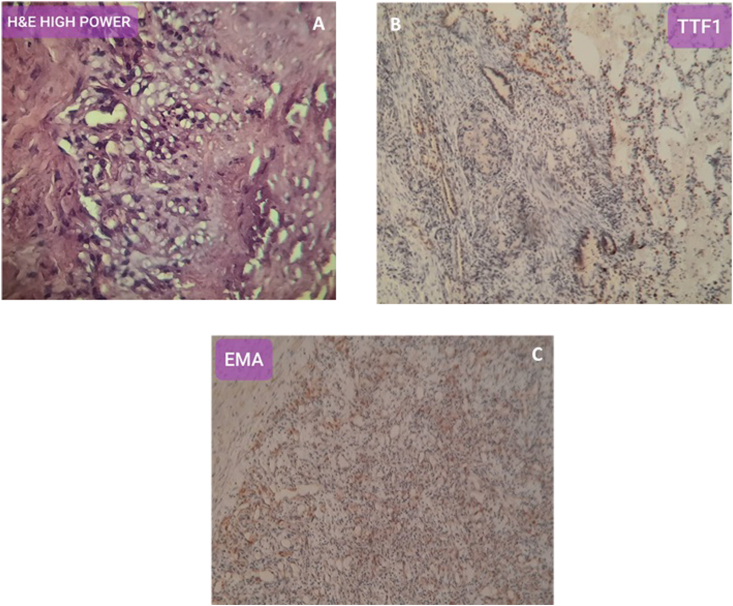
Pulmonary sclerosing hemangioma was confirmed by microscopic examination and immunohistochemical study. A. Neoplastic proliferation made-up of round to polygonal cells containing oval nuclei embedded within sclerotic highly vascularized stroma. B. Positive TTF1 staining. C. Positive EMA staining.

Histopathological examination reveals tumoral proliferation made - up of round to polygonal cells containing oval nuclei embedded within sclerotic highly vascularized stroma (Fig. 4A).

These results were compatible with sclerosing pneumocytoma (SP), and peri-vascular epithelioid cells tumor (PEComa). The immunohistochemical study revealed the following results: thyroid transcription factor-1 (TTF1) was positive on a few numbers of proliferated cells (Fig. 4B), Vimentin was positive, epithelial membrane antigen (EMA) was mild-focal positive (Fig. 4C), CD34 and HMB45 were negative. These findings confirmed the presence of SP.

Fourteen months postoperatively, the patient was in a good health with no clinical or radiological evidence of recurrence.

## 3 Discussion

SP is a rare benign neoplasm of the lung. It was first described by Liebow and Hubell in 1956 [1]. The peak age incidence is at fifth decade, with a female to male ratio of 5:1 in this patient group [5]. To the best of our knowledge, the youngest reported patient with SP in the literature was 4 years old [4]. But here we report a one-year-old girl with SP. Most of the SP patients are clinically asymptomatic, but fever had been mentioned previously in the literature as a rare predominant symptom [7]. For our patient, hemoptysis was the main symptom. SP is histologically characterized by the presence of hemorrhagic, papillary, solid, or sclerotic areas, and the majority of tumors will display an admixture of two or more of these patterns in differing proportions [5]. The immunohistochemical examination in more than 90% of SP cases revealed that the tumor cells were immunopositive for TTF1 and EMA [8], as also shown in our case. SP is often detected as a round, well-defined homogenous mass on routine chest radiography. Marginal pseudocapsule, overlying vessel, air gap, and halo sign are some radiologic signs that could be observed on the chest CT scan. Calcification may also be seen [9]. SP can rarely be bilateral [10]. Although the tumor is generally considered to be a benign lesion, SP can cause hilar or mediastinal lymphadenopathy or lung-to-lung metastasis, especially when the tumor is large [3]. Recurrence had been mentioned previously in a study that showed a recurrence 10 years after resection [11]. SP is usually diagnosed and treated by thoracotomy. The diagnosis is made by the histopathological and immunohistochemical examination. Patients with SP have an excellent prognosis with no need for additional treatment after surgical resection [12]. It may be necessary to dissect the lymph nodes in cases of a large SP or in the presence of enlarged lymph nodes that may be due to metastasis. The prognosis is not strongly influenced in such cases [13].

In conclusion, we present a case of Sclerosing pneumocytoma in a 1-year-old girl with hemoptysis. Because SP mostly presents in middle aged-women, this case highlights the importance of considering SP in young children. Also, while almost all cases are asymptomatic or with nonspecific symptoms, this case shows why it's important to consider the SP in the differential diagnosis of hemoptysis.

## 4 Informed consent

Written informed consent was obtained from the patient's parents for publication of this case report and accompanying images. A copy of the written consent is available for review by the Editor-in-Chief of this journal on request.

## Provenance and peer review

Not commissioned, externally peer-reviewed.

## Funding

There was no funding.

## Ethical Approval

No ethical approval was needed.

## Author contribution

IA: reviewed the literature, wrote the abstract, and the discussion; AB: reviewed the literature, wrote the case presentation, designed the figures and provided the captions of the figures; OD: reviewed the literature, wrote the introduction, and conclusion; FA and LH: worked on patient diagnosis, did the histopathological study, did the follow-up, and helped writing the discussion; BD: lead the surgical team, did the operation, reviewed the case, and did the grammar check-up.

## Consent

Written informed consent was obtained from the patient’s parents for publication of this case report and accompanying images. A copy of the written consent is available for review by the Editor-in-Chief of this journal on request.

## Registration of Research Studies

N/A.

## Guarantor

Mr. Obada Daaboul

## Declaration of competing interest

All of the authors declared that they have no conflict of interest.
